# Validation of a deep learning-based system for sleep staging, arousal detection, and respiratory event scoring in atrial fibrillation patients

**DOI:** 10.1007/s44470-026-00145-0

**Published:** 2026-08-03

**Authors:** Susana Sousa, Carlos Teixeira, Thóra Sigmarsdóttir, Eysteinn Finnsson, Jón Ágústsson, Sindri Sigþórsson, Snorri Bjarkason, Marta Drummond, António Bugalho

**Affiliations:** 1 Sleep Unit, CUF Tejo Hospital and CUF Descobertas Hospital, Lisbon, Portugal; 2 Pulmonology Department, CUF Tejo Hospital, Lisbon, Portugal; 3 NOVA Medical School, Lisbon, Portugal; 4 Comprehensive Health Research Center (CHRC), Lisbon, Portugal; 5 Nox Research, Nox Medical, Katrínartún 2, 105 Reykjavík, Iceland; 6 H²M - Health and Human Movement Unit, Polytechnic University of Health, CESPU, Vila Nova de Famalicão, Portugal; 7 Sleep and Non-Invasive Unit, Centro Hospitalar E Universitário São João, Porto, Portugal; 8 Faculty of Medicine, University of Porto, Porto, Portugal

**Keywords:** Sleep apnea, Atrial fibrillation, Sleep-disordered breathing, Machine learning, Sleep stage, Polysomnography

## Abstract

**Purpose:**

Obstructive sleep apnea is a common yet underdiagnosed sleep disorder in patients with atrial fibrillation (AF). Although polysomnography (PSG) is the gold standard, its costs and complexity limit routine use. Home sleep apnea testing (HSAT) is a more accessible alternative, but it lacks electroencephalography (EEG), limiting direct assessment of sleep stages and arousals. This study evaluates the performance of DeepRESP, an automated scoring solution that infers sleep stages and arousals using respiratory inductance plethysmography (RIP) signals and scoring respiratory events, in a cohort of patients with AF.

**Methods:**

In this prospective cohort study, consecutive AF patients underwent ambulatory type II PSG. Automated respiratory event scoring, sleep staging and arousal identification were performed with DeepRESP using only HSAT signals. Performance metrics included epoch-level positive and negative percent agreement, complemented by concordance analyses for the apnea–hypopnea index (AHI), arousal index (ArI), and total sleep time (TST), using Bland–Altman analysis and intraclass correlation coefficients (ICC), with manually scored PSG as reference.

**Results:**

A total of 88 patients (67.0% male) were included. Women were older than men (66.7 ± 7.2 vs. 61.27 ± 10.3 years, *p* = 0.015). Epoch-level overall percent agreement (OPA) with manual scoring was 0.91 for Wake, 0.95 for rapid eye movement sleep (REM), and 0.87 for non-rapid eye movement sleep (NREM). Arousal detection OPA was 0.80. Strong agreement was observed for the apnea–hypopnea index (ICC = 0.92), TST (ICC = 0.82), and ArI (ICC = 0.83).

**Conclusion:**

In AF patients, DeepRESP reliably estimates sleep architecture, arousals, and sleep apnea severity from HSAT signal, offering a scalable, EEG-independent diagnostic approach.

**Brief summary:**

**Current knowledge/study rationale:**

Obstructive sleep apnea is highly prevalent and often underdiagnosed in atrial fibrillation patients, yet standard home testing lacks the neurophysiological channels necessary to detect arousals and accurate sleep architecture. This study was conducted to validate a deep learning-based system capable of inferring these critical metrics exclusively from home sleep testing signals, improving diagnostics performance within this specific cardiovascular population.

**Study impact:**

The findings demonstrate that artificial intelligence-driven analysis of breathing patterns achieves high concordance with polysomnography for key diagnostic indices, including arousal index, total sleep time and apnea severity via the apnea–hypopnea index. This provides a scalable, precise diagnostic pathway that bypasses the confounding effects of arrhythmias and cardiac medications, facilitating improved management of sleep-disordered breathing in atrial fibrillation patients.

## Introduction

Obstructive sleep apnea (OSA) is highly prevalent among patients with atrial fibrillation (AF) [1, 2] and significantly compromises the efficacy of rhythm control interventions [3]. Current patient assessment involves a thorough clinical evaluation, the use of validated sleep questionnaires, and comprehensive sleep studies to confirm the diagnosis of sleep-disordered breathing (SDB) [4]. However, the optimal screening modality for SDB in AF patients has yet to be established. While in-laboratory manually scored polysomnography (PSG) sleep recordings are the gold standard for the diagnosis of SDB, through the monitoring of brain activity (electroencephalography, EEG), eye movements (electrooculography, EOG), muscle activity (electromyography, EMG), heart rhythm (electrocardiography, ECG), respiratory airflow and effort, pulse oximetry, body position, and snoring [4], the associated costs and logistical challenges limit its scalability for routine AF diagnosis and management.

Consequently, home sleep apnea testing (HSAT) has emerged as a more feasible alternative. However, standard HSAT primarily monitors respiratory parameters and oxygen saturation levels but does not measure neurophysiological channels (EEG, EOG, and EMG), necessary to identify sleep stages and arousals [4]. These limitations diminish the effectiveness of diagnosing SDB beyond OSA. Crucially, it may lead to the underdiagnosis of OSA in patients with limited sleep duration or those exhibiting a high proportion of hypopneas associated with arousals rather than significant oxygen desaturation [5]. Furthermore, HSAT cannot adequately diagnose other sleep disorders common in AF patients, such as insomnia [6].

The diagnostic challenge arises from the overlapping nonspecific symptoms between AF and OSA [7]. Symptoms such as nocturnal dyspnea, fatigue, exercise intolerance, and cognitive alterations are frequently attributed solely to underlying cardiac disease and AF, leading to underdiagnosis of OSA [8–10]. Conversely, symptoms primarily driven by AF—such as nocturnal palpitations and sleep disruption—may be misattributed to OSA, potentially delaying appropriate cardiac evaluation. On the other hand, unrecognized OSA perpetuates and exacerbates AF through several well-characterized pathophysiological mechanisms, including intermittent hypoxemia, increased sympathetic activation, and cardiac structural remodeling [11, 12]. Given the substantial impact that OSA diagnosis has on AF outcomes, including increased recurrence rates after cardioversion, reduced efficacy of antiarrhythmic medications [13], and higher cardiovascular mortality [14], an accurate diagnosis of these patients becomes imperative.

Breathing patterns vary across sleep–wake states due to changes in neural control and muscle activity. During wakefulness, respiration is irregular and influenced by voluntary behaviors. In NREM sleep, breathing becomes stable and metabolically regulated. In REM sleep, respiration is again irregular, but skeletal muscle atonia alters thoracoabdominal mechanics and the resulting movement patterns. These state-dependent differences are reflected in RIP signals, enabling differentiation between wakefulness, NREM, and REM sleep. In addition, arousals are associated with a ventilatory response, producing detectable perturbations in respiratory signals.

The application of precision medicine principles in this context involves developing personalized diagnostic strategies that account for patient-specific sleep phenotypes. Specifically, identifying arousals as potential arrhythmogenic triggers via EEG is fundamental in AF patients. In this study, we leverage artificial intelligence techniques to model the interaction between respiratory patterns and sleep physiology. By identifying sleep stages and arousal events from respiratory signals alone, this approach aims to provide a simplified, faster, and cost-effective diagnostic pathway without compromising the accuracy of gold-standard metrics.

## Material and methods

### Study objective and analysis approach

The objective of this study was to validate the performance of DeepRESP, a cloud-based software as a medical device (SaMD) developed by Nox Medical (FDA 510(k) K241960) [15], in patients with AF, with a specific focus on its ability to support HSAT-based sleep apnea assessment. Although the recordings analyzed in this study were acquired as level II PSG, DeepRESP processed each study using only the subset of physiological signals typically available in HSAT recordings. Manually scored PSG recordings were used as the reference standard for validation.

DeepRESP provides automated analysis of sleep study data, including sleep staging, arousal detection, respiratory event scoring, and the calculation of diagnostic indices such as the AHI. In the current study, the specific tasks of sleep staging and arousal detection were performed using Nox BodySleep 2.0 (NBS2) [16] which is integrated into the DeepRESP software. The NBS2 algorithm relies exclusively on thoracic and abdominal respiratory inductance plethysmography (RIP) signals rather than EEG. By analyzing breathing patterns, it estimates sleep state and arousal probability, enabling the derivation of clinically relevant sleep and arousal metrics, including total sleep time (TST) and the arousal index (ArI), from HSAT-compatible signals.

This arousal detection capability further enables the identification and scoring of hypopneas that terminate in an arousal without an associated oxygen desaturation, consistent with gold standard PSG-based scoring practices.

Prior to its application in this study, the clinical performance of DeepRESP was validated in a large retrospective study using manually scored sleep recordings originating from sleep clinics across the United States. For the validation of Type III (without EEG) scoring capabilities, the study utilized a total of 3488 sleep recordings collected during standard clinical care for patients with suspected sleep disorders. The test population was highly diverse and representative of general clinical practice, including patients from urban, suburban, and rural areas with broad racial and ethnic diversity. Demographically, the validation cohort included 33% females and spanned all adult age categories (22 to > 65 years) and BMI groups (< 25, 25–30, and > 30 kg/m^2^). Against this generalized clinical population, the algorithm demonstrated non-inferiority or superiority to established predicate devices in sleep state estimation, respiratory event scoring, and arousal detection, thus fulfilling the predefined clinical performance criteria for its FDA 510(k) clearance.

All DeepRESP outputs evaluated in this study were generated fully automatically, without any manual editing or scorer intervention. Respiratory events, including apneas and hypopneas, were scored algorithmically using only HSAT-compatible physiological signals and were used to derive clinical parameters, including the AHI. It should be noted that DeepRESP does not score respiratory effort-related arousals (RERAs). The study design reflects the intended clinical use of DeepRESP in home sleep apnea testing and allows assessment of its ability to reproduce gold standard PSG-referenced diagnostic measures.

The following are the definitions of scoring approaches:

- Manual PSG scoring refers to gold standard PSG scoring using the full set of PSG signals, including EEG, EOG, EMG, oximetry, nasal pressure airflow, and thoracic and abdominal RIP signals. Sleep stages, arousals, oxygen desaturations, and respiratory events (apneas and hypopneas) are identified and scored by a certified sleep physician and a sleep technician using standard criteria defined by the American Academy of Sleep Medicine [17, 18].
- Automated HSAT scoring refers to fully automated analysis performed by DeepRESP using only a subset of physiological signals typically available in HSAT recordings, specifically oximetry, nasal pressure airflow, and RIP signals. In this approach, sleep stages, arousals, desaturations, and respiratory events are inferred algorithmically without access to EEG or other neurophysiological signals and without human scorer intervention.

### Signal acquisition parameters

All physiological signals analyzed in this study were acquired during level II PSG recordings using the Nox A1 portable monitor (Nox Medical, Reykjavik, Iceland). The RIP signals (thoracic and abdominal) were sampled at 25 Hz, the nasal pressure airflow signal at 25 Hz, and the oximetry signal at 1 Hz—sampling rates consistent with those employed in standard in-laboratory PSG systems and within the specifications recommended by the AASM [19] for HSAT-compatible signals. As the same device and acquisition parameters were used for both the PSG reference and the HSAT-compatible signal subset processed by DeepRESP, no differences in sampling rate or filter settings existed between the two scoring approaches evaluated in this study.

### Study population

Consecutive patients aged between 18 and 75 years old with AF were evaluated at a cardiology outpatient unit and referred for sleep assessment and PSG. All patients with AF were invited to participate, regardless of their OSA risk profile.

Exclusion criteria included a previous diagnosis of OSA, unstable coronary artery disease, myocardial infarction, or percutaneous coronary intervention within 3 months prior to the study.

Participants were recruited at CUF Tejo Hospital between January 2 and November 3, 2023. The study was approved by the local ethics committee of CUF Tejo Hospital and by the NMS|FCM (CEFCM) Ethics Committee. All participants provided their informed consent. All participants were referred for sleep assessment, which included a thorough medical history and physical examination and type II PSG recording. Clinical evaluation included measurement of height, weight, and body mass index (BMI). The Epworth Sleepiness Scale (ESS) was used to assess daytime hypersomnolence. Ambulatory PSG was performed using a Nox A1s PSG system (Nox Medical, Reykjavik, Iceland). The following signals were recorded simultaneously: 6-channel EEG (leads: F3A2, F4A1, C3A2, C4A1, O1A2, and O2A1), left and right EOG (LOC, ROC), and submental EMG (3 unipolar leads). Additionally, nasal airflow pressure was measured with a nasal cannula, respiratory effort was assessed using thoracic and abdominal inductive plethysmography, and snoring was recorded with a built-in microphone. Body position and activity were measured with the internal 3-axis, ± 2-g accelerometer with a sampling frequency of 10 Hz. Oximetry and sound intensity (dB) were measured. Manual sleep staging and respiratory scoring were performed by a certified sleep physician and a sleep technician using standard criteria defined by the American Academy of Sleep Medicine [18]. The AHI was defined as the combined number of apneas and hypopneas per hour of sleep. The hypopnea criteria used was 1 A, defined as a ≥ 30% decrease in airflow for at least 10 s, followed by a ≥ 3% decrease in saturation or the event is associated with an arousal [18].

### Statistical analysis

Validation of the DeepRESP system in the AF cohort involved analysis at both the epoch level and clinical parameter level to determine concordance between manual PSG and automatic HSAT scoring.

Diagnostic performance was assessed using positive percentage (PPA), negative percentage (NPA), and overall percentage agreement (OPA) for the detection of arousals, apneas, hypopneas, and respiratory events (where a respiratory event was defined as either an apnea or a hypopnea), as well as for sleep stage classification (wake, non-rapid eye movement [NREM] sleep, and rapid eye movement [REM] sleep). Bootstrapping was applied with 10,000 iterations to derive 95% confidence intervals (CI) for all epoch-level performance metrics.

Percentage agreement for clinical parameters (AHI, TST, hypopnea index (HI), Apnea Index (AI), and ArI), was evaluated using Bland–Altman analysis and Intraclass Correlation Coefficients (ICC). Bland–Altman analysis was used to estimate the bias (mean difference) and the 95% limits of percentage agreement (LoA) between manual PSG scoring and automated HSAT scoring. Bland–Altman plots are presented for selected key clinical parameters (AHI, TST, and ArI), while numerical Bland–Altman statistics are reported for all parameters. Bootstrapping with 10,000 iterations was applied to obtain 95% confidence intervals for all bias, LoA, and ICC estimates.

## Results

### Patients’ characteristics

A total of 88 consecutive patients with AF (59 male, 29 female) were included. The mean age was significantly higher in women than in men (66.72 ± 7.181 vs. 61.27 ± 10.347 years, *p* = 0.015). Paroxysmal AF was present in 66 patients, whereas 12 patients had persistent AF. The mean BMI was 30 ± 4.8 kg/m^2^. Most patients presented with additional comorbidities, predominantly obesity (50.0%) arterial hypertension (46.5%) and diabetes mellitus (17.8%). The mean ESS score was 6.3 ± 4.39, and only 23.86% of patients exhibited clinically significant daytime sleepiness, defined as an ESS ≥ 10. A history of snoring was reported in 64.65% of the study population.

No significant differences were observed between genders regarding body mass index (BMI), prevalence of hypertension, diabetes mellitus or daytime somnolence (Table 1). The population was predominantly non-sleepy, with only 23.5% of patients presenting with an ESS score ≥ 10. Our study revealed a 100% prevalence of OSAS in the cohort of AF patients. All participants had an AHI ≥ 5 per hour (mild OSA) and 80% of patients had an AHI of ≥ 15 per hour (moderate to severe OSAS).

**Table 1:** Baseline characteristics of the study population according to gender

Variable	Male (*n* = 59)	Female (*n* = 29)	*p*-value
Demographics			
Age, years	61.27 ± 10.347	66.72 ± 7.181	0.015
BMI	30.441 ± 4.408	29.806 ± 5.384	0.564
Epworth Scale	6.653 ± 4.389	6.103 ± 3.726	0.765
Risk factors			
Hypertension, n (%)	29 (49.2%)	15 (55.6%)	0.646
Diabetes, n (%)	10 (16.9%)	5 (18.5%)	1,000

### Performance and epoch-level agreement

The epoch-level agreement between manual PSG and automatic HSAT scoring is summarized in Table 2, which reports PPA, NPA, and OPA together with their 95% CI. The system identified arousals with a PPA of 0.72 [95% CI: 0.68, 0.75], a NPA of 0.84 [95% CI: 0.81, 0.86], and an OPA of 0.80 [95% CI: 0.78, 0.81]. For sleep staging, the system achieved a class OPA of 0.91 [0.89, 0.93] for Wake, 0.95 [CI: 0.94, 0.95] for REM, and 0.87 [CI: 0.85, 0.88] for NREM. Performance for respiratory event detection yielded an overall OPA of 0.94 [0.92, 0.95] for apneas, 0.78 [0.77, 0.79] for hypopneas, and 0.81 [0.80, 0.83] for respiratory events.

**Table 2:** Epoch-level agreement between manual PSG scoring and automatic HSAT scoring for arousal, sleep stages, and respiratory events. Agreement is quantified using PPA, NPA, and OPA, with their 95% CI shown in brackets

Epoch	PPA	NPA	OPA
Arousal	0.72 [0.68, 0.75]	0.84 [0.81, 0.86]	0.80 [0.78, 0.81]
Sleep–wake	0.81 [0.78, 0.84]	0.93 [0.90, 0.94]	0.91 [0.89, 0.93]
Sleep-REM	0.77 [0.73, 0.81]	0.98 [0.97, 0.98]	0.95 [0.94, 0.95]
Sleep-NREM	0.89 [0.86, 0.91]	0.82 [0.80, 0.84]	0.87 [0.85, 0.88]
Apnea	0.86 [0.81, 0.90]	0.95 [0.94, 0.96]	0.94 [0.92, 0.95]
Hypopnea	0.67 [0.64, 0.71]	0.84 [0.81, 0.86]	0.78 [0.77, 0.79]
Respiratory-event	0.82 [0.78, 0.85]	0.81 [0.78, 0.83]	0.81 [0.80, 0.83]

*CI* confidence interval, *NPA* negative percent agreement, *NREM* non-rapid eye movement, *OPA* overall percent agreement, *PPA* positive percent agreement, *REM* rapid eye movement

The agreement between manual PSG scoring and automatic HSAT scoring for clinical diagnostic parameters (AHI, TST, hypopnea index (HI), apnea index (AI), and ArI) was evaluated using Bland–Altman analysis and ICC.

**Fig. 1:**
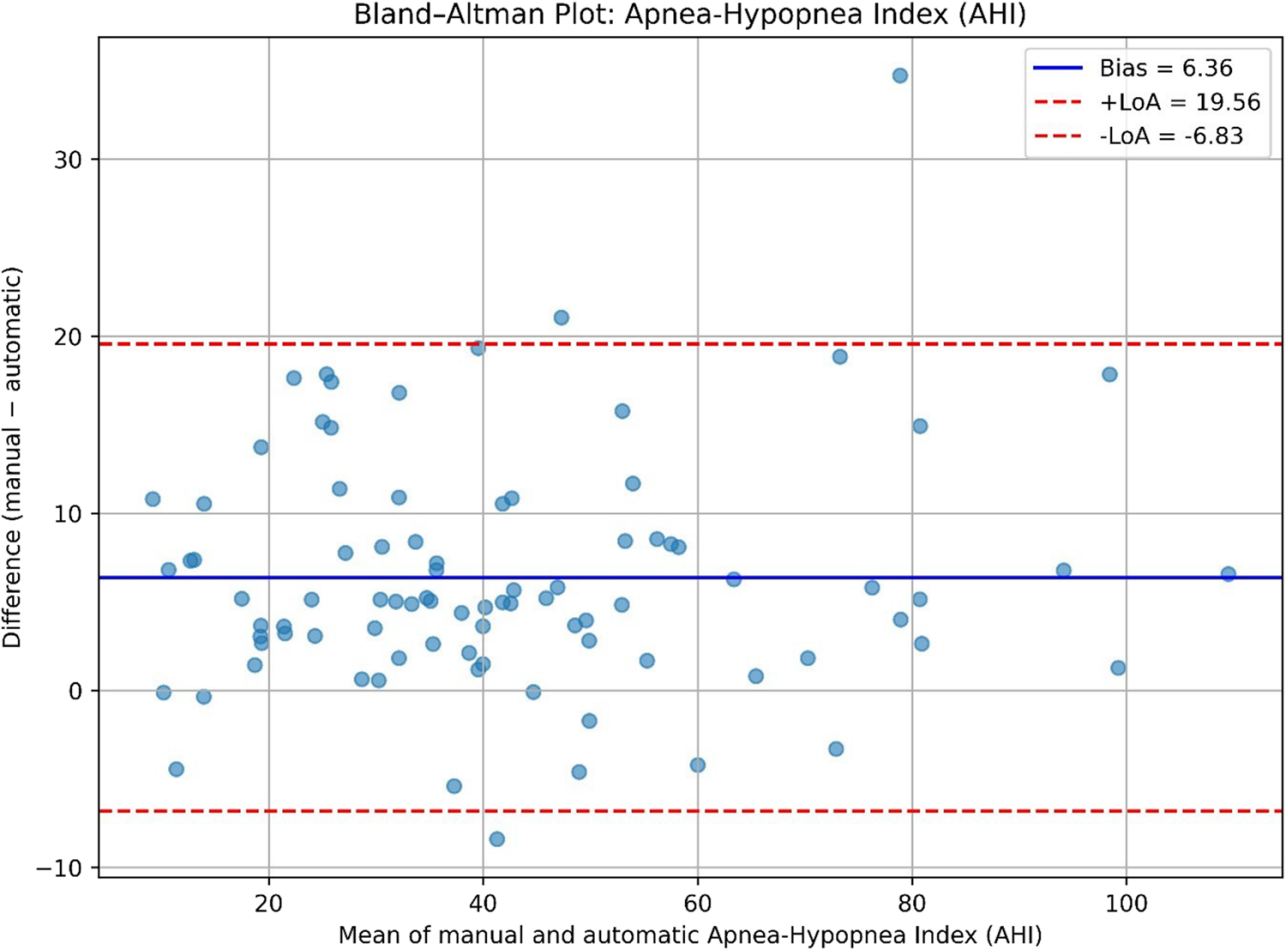
Bland–Altman plot showing the agreement between the manual PSG scoring and the automatic HSAT scoring for the AHI. Each data point represents one sleep study. The plot shows the mean difference (bias, blue line) and the 95% limits of agreement (dashed red lines)

The primary metric, AHI, showed excellent correlation with an ICC of 0.92 [95% CI: 0.88, 0.95]. Bland–Altman analysis revealed a mean bias of 6.36 [95% CI: 5.02, 7.79], with LoA ranging from − 6.83 to 19.56 (Fig. 1). The observed AHI bias was primarily driven by the Hypopnea Index (bias: 5.45 events/hour), whereas the Apnea Index demonstrated high stability (bias: 0.91 events/hour).

**Fig. 2:**
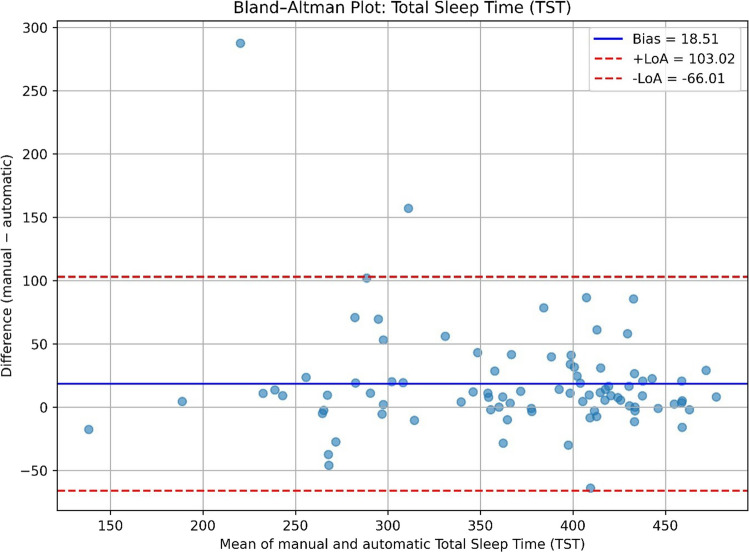
Bland–Altman plot showing the agreement between the manual PSG scoring and the automatic HSAT scoring for the ArI. Each data point represents one sleep study. The plot shows the mean difference (bias, blue line) and the 95% limits of agreement (dashed red lines)

**Fig. 3:**
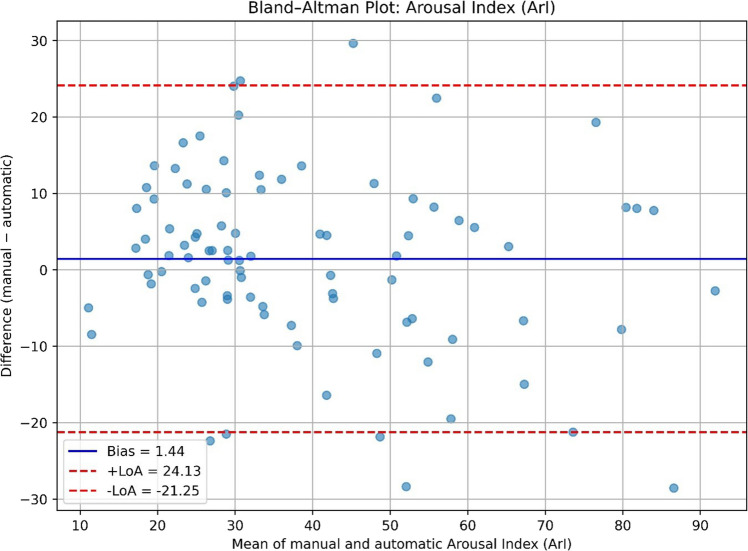
Bland–Altman plot showing the agreement between the manual PSG scoring and the automatic HSAT scoring for the TST. Each data point represents one sleep study. The plot shows the mean difference (bias, blue line) and the 95% limits of agreement (dashed red lines)

The ArI exhibited a low mean bias of 1.44 [−1.02, 3.87] with an ICC of 0.83 (Fig. 2), although individual variance increased at higher arousal frequencies. TST showed a mean bias of 18.51 min [10.30, 28.08] with an ICC of 0.82 (Fig. 3). Comprehensive statistics for all parameters are summarized in Table 3.

**Table 3:** Agreement analysis between manual PSG scoring and the automatic HSAT scoring, showing ICC, bias, and ± 95% LoA for AHI, TST, and AI

Class	ICC (2, 1)	Bias	LoA −	LoA +
AHI	0.92 [0.88, 0.95]	6.36 [5.02, 7.79]	− 6.83 [− 9.31, − 4.29]	19.56 [15.85, 23.36]
ArI	0.83 [0.75, 0.89]	1.44 [− 1.02, 3.87]	− 21.25 [− 25.85, − 16.26]	24.13 [19.92, 27.81]
TST	0.82 [0.66, 0.93]	18.51 [10.30, 28.08]	− 66.01 [− 94.74, − 36.22]	103.02 [61.91, 146.71]
AI	0.91 [0.83, 0.98]	0.91 [− 0.44, 2.65]	− 13.98 [− 19.72, − 5.99]	15.80 [6.16, 24.55]
HI	0.82 [0.68, 0.89]	5.45 [3.55, 7.14]	− 11.64 [− 18.47, − 6.12]	22.54 [17.85, 27.39]

*AHI* apnea–hypopnea index, *AI* apnea index, *ArI* arousal index, *HI* Hypopnea index, *ICC* intraclass correlation coefficients, *LoA* limits of agreement, *TST* total sleep time

## Discussion

This study evaluated a novel artificial intelligence-based system (DeepRESP), providing evidence that it accurately estimates sleep architecture, arousals, and sleep apnea severity from HSAT signals in AF patients [16]. SDB is highly prevalent among patients with AF and is a recognized factor in reducing the efficacy of rhythm control strategies [14, 20]. Current assessment relies on thorough clinical evaluation, application of sleep-related questionnaires, and objective sleep studies to establish a definitive diagnosis [4]. While PSG remains the gold standard for SDB diagnosis, its limited availability, high cost, and logistical complexity limit its widespread use, underscoring the need for accessible, reliable alternative screening and diagnostic strategies [4, 16, 21].

Diagnosing OSA in AF patients is particularly challenging, as this population is often pauci-symptomatic regarding sleep-related complaints [22, 23]. Nevertheless, systematic screening for OSA in AF patients is essential, given the significant implications of untreated OSA on the management and outcomes of AF itself [20, 24]. There is a growing need to apply precision medicine principles in the diagnosis of SDB, ensuring accurate characterization of respiratory events and associated arousals [25, 26]. This level of diagnostic precision is essential, as arousals are known to significantly influence autonomic nervous system activation, which may have critical implications for cardiovascular outcomes [27, 28].

Simplified sleep studies typically do not provide the level of physiological detail required for a precise diagnosis of SDB or for an accurate assessment of its severity, potentially leading to underestimation of this disease and suboptimal management [4, 21]. A key challenge in reduced-channel systems is that they rely on indirect physiological markers whose relationship to sleep state, arousals, and respiratory events may vary across clinical populations [16]. The critical issue is therefore not whether a signal is indirect per se, but whether the physiological coupling between the measured signal and the target phenomenon is strong, stable, and clinically validated in the population of interest. In routine clinical practice, when utilizing simplified sleep studies, it is essential to ensure that the utilized technology is appropriate for the targeted population [4]. In one study involving patients with AF, it was found that the inclusion of arousal-based criteria substantially improves OSA detection and influences severity classification, highlighting that arousal-inclusive hypopnea scoring provides a more accurate assessment and supports personalized treatment strategies [5]. In contrast, a recent evaluation of peripheral arterial tonometry–based home testing in patients with AF demonstrated only slight to fair agreement with PSG for AHI-based severity classification, with a tendency to overestimate OSA severity [29]. These findings underscore how AHI estimation may vary substantially depending on the underlying physiological signal source and algorithmic assumptions, particularly in rhythmically unstable populations. Previous efforts to infer sleep stages without EEG have used different combinations of physiological signals, such as respiratory and cardiac [30], respiratory, cardiac and movement signals [31]. However, incorporating cardiac-derived features may present challenges in individuals with cardiovascular conditions, especially those with rhythm disorders such as AF. In this group, irregular heart rate patterns and altered autonomic regulation can reduce the stability and reliability of cardiac-based markers for sleep staging, potentially limiting algorithm accuracy and generalizability.

Our findings demonstrate that EEG-independent sleep scoring is feasible even in an OSA high-risk AF population, challenging the traditional assumption that neurophysiological channels are indispensable for reliable sleep architecture assessment. This aligns with recent large-scale validation data showing that the DeepRESP algorithm achieves strong agreement with expert EEG-based scoring, specifically reaching an ICC of 0.82 for TST and 0.83 for the ArI [16]. By utilizing RIP signals, the algorithm leverages the distinctive breathing dynamics of different sleep states—ranging from the metabolic-driven monotony of NREM to the high variability and intercostal muscle atonia characteristic of REM sleep [16].

A significant milestone of this approach is the automated detection of arousals via breathing patterns [16]. Arousals induce a transient ventilatory response, often characterized by a gasp-like reflex or “ventilatory response to arousal” (VRA), which is described as a physiological response that can be measured and quantified [16, 32]. Capturing these events is fundamental for AF patients, as the algorithm's ability to detect arousals, beyond those linked to respiratory events, ensures a comprehensive assessment of sleep fragmentation [16]. Furthermore, by focusing exclusively on RIP signals, this diagnostic approach avoids confounding factors common in cardiogenic-based sleep staging, such as the effects of cardiac medications (e.g., beta-blockers) or underlying arrhythmias, which frequently complicate analysis in the AF population [33–35]. In addition, as signal acquisition was performed under technologist supervision,

RIP signal quality may have been superior to that typically observed in real-world unattended HSAT, where sensor displacement and suboptimal belt positioning are more frequent, possibly affecting algorithm performance [36, 37]. Additionally, while current clinical HSAT practice typically incorporates multiple physiological channels including airflow and oximetry, the NBS2 component of DeepRESP was specifically designed to operate exclusively on RIP signals. Although this may not reflect standard multi-channel HSAT configurations, this design choice is grounded in the physiological rationale that thoracic and abdominal respiratory effort signals independently encode sleep-state and arousal-related information [16]. Respiratory effort measurement is a mandatory signal in both PSG and HSAT systems, and in our case, we used RIP, knowing that it is robust to cardiac arrhythmia, and avoids the confounding effects of cardiac medications common in AF populations [33–35]. This potential for RIP-only scoring represents a critical advancement in applying precision medicine principles to sleep diagnostics without the complexity of in-lab PSG.

### Limitations

This study has several limitations. First, the sample size was relatively small and limited to a single tertiary cardiology center, which may introduce referral bias and restrict generalizability. Second, although the recordings were acquired in an ambulatory setting, they were performed as Level II PSG [4]. Therefore, while DeepRESP processed the data using only HSAT-compatible signals, the recordings were still obtained under more controlled conditions than fully unattended home testing [36]. Algorithm performance may vary in truly unattended HSAT settings, where technical failures and signal loss are more likely due to suboptimal sensor placement and displacement during the night [36, 37].

Furthermore, while concordance was high for AHI and TST, the ArI showed wider limits of agreement, suggesting that respiratory derived arousal estimates, although clinically informative, may not fully substitute EEG based arousal scoring for granular characterization of sleep fragmentation. A further limitation concerns the potential influence of comorbid conditions on automated sleep analysis. Certain comorbidities frequently observed in AF patients—including heart failure, Cheyne-Stokes respiration, and hypoventilation syndromes—may produce breathing patterns that overlap with or mimic obstructive events, potentially affecting the accuracy of respiratory event classification and sleep parameter estimation. We acknowledge that these comorbidities may affect algorithm performance and that their identification has direct therapeutic implications, including the possible need for respiratory assist devices such as bilevel PAP or adaptive servo-ventilation. We also note explicitly that DeepRESP has been trained and validated on diagnostic studies only, and that its performance in patients using respiratory support has not been assessed. In addition, as signal acquisition was performed under technologist supervision, RIP signal quality may have been better than in real-world unattended HSAT, where sensor displacement and suboptimal belt positioning are more common and may affect algorithm performance. Therefore, further validation in fully unattended HSAT settings is warranted.

Finally, the cohort consisted exclusively of patients with AF, and the findings may not be generalizable to populations in whom breathing dynamics and sleep-related respiratory patterns differ substantially, including patients with significant cardiorespiratory disease, respiratory muscle weakness due to neuromuscular conditions, or severe insomnia, where PSG is generally preferred over HSAT [4].

## Conclusions

In conclusion, this study validates the performance of DeepRESP, an AI-based system for automated sleep staging, arousal detection, and respiratory event scoring in patients with atrial fibrillation. The system demonstrated high concordance with gold-standard manual PSG, with strong agreement for core diagnostic indices, including an ICC of 0.92 for AHI and 0.82 for TST.

By enabling the identification of arousal-associated respiratory events without EEG, this approach addresses a major limitation of conventional HSAT and reduces the recognized risk of underdiagnosing SDB in minimally symptomatic AF patients. Furthermore, the reliance on breathing signals ensures diagnostic stability by bypassing common confounding factors inherent to cardiogenic signals such as arrhythmia and rate-control therapy.

While the agreement for the ArI showed wider limits of agreement and systematic differences that should be considered in clinical interpretation, these findings support the use of HSAT as a scalable and precise pathway for AF-focused sleep assessment. This represents a critical advancement toward precision medicine in sleep diagnostics, warranting further validation in fully unattended home recordings to evaluate its long-term impact on clinical management and cardiovascular outcomes.

## Data Availability

Data that support the findings of this study are not publicly available due to privacy reasons but are available from the corresponding author upon reasonable request.
